# Effects of Soil Properties and Seasonal Variations on Microbial Communities in Constructed Wetlands

**DOI:** 10.1007/s00248-025-02564-7

**Published:** 2025-06-12

**Authors:** Ting-Kai Chen, Yo-Jin Shiau

**Affiliations:** 1 Dept. of Bioenvironmental Systems Engineering, National Taiwan University, Taipei, Taiwan; 2 Agricultural Net-Zero Carbon Technology and Management Innovation Research Center, National Taiwan University, Taipei, Taiwan

**Keywords:** Constructed wetlands, Microbial diversity, Next generation sequencing

## Abstract

Constructed wetlands (CWs) are important ecosystems with numerous benefits such as wastewater treatment, wildlife habitat protection, and stormwater remediation. However, the development of soil microbial communities in CWs over time remains understudied. This study comprehensively investigates microbial diversity and community composition in three constructed wetlands, focusing on the influence of wetland age, soil depth, and environmental factors. The results indicate that both soil depth and seasonal variations significantly affect alpha diversity, particularly in surface soils. The predominant microbial communities, including nitrifying and denitrifying communities, were identified across the studied wetlands. Moreover, sulfate-rich conditions may promote sulfur autotrophic denitrification. Redundancy analysis and multiple linear regression highlighted the distinction between autotrophic and heterotrophic denitrifiers. Soluble organic carbon was identified as a major factor influencing heterotrophic denitrifying bacteria, while sulfate and nitrate levels were more closely associated with autotrophic denitrifying bacteria. Overall, these findings provide valuable insights into microbial community dynamics in CWs and can help optimize wetland management strategies for improved nutrient removal efficiency.

**Supplementary Information:**

The online version contains supplementary material available at 10.1007/s00248-025-02564-7.

## Introduction

Wetlands are renowned for their ecosystem services, such as purifying polluted water, remediating stormwater, and supporting diverse wildlife communities. Constructed wetlands (CWs) are engineered systems designed to achieve one or more of these ecosystem services, particularly for improving water quality, by mimicking the physicochemical conditions of natural wetlands [1–3].

Soil microorganisms play crucial roles in various biogeochemical processes, facilitating the decomposition of excess nutrients in wetland ecosystems under both aerobic and anaerobic soil conditions and are the foundation of the wetland ecosystems [3–6]. For example, studies have shown that microbial nitrification and denitrification processes regulate approximately 80–90% of the total nitrogen in CWs [7, 8]. Additionally, phosphorus-accumulating organisms may absorb phosphate from wastewater under alternating aerobic and anaerobic conditions [9, 10].

However, the nutrient removal as well as other ecosystem functions of CWs can differ due to design factors that alter soil physicochemical properties and microbial compositions [11]. Studies have shown that factors such as soil depth gradients [12], water depth [13], soil organic carbon and nitrogen concentrations, soil water content [14–16], and pH [17] can substantially influence microbial communities. Furthermore, soil microbial communities are likely to shift over time, as the soil total organic carbon (TOC) in wetlands may gradually increase with wetland age [18, 19]. Thus, understanding the composition and diversity of microbial communities and their relationships with environmental parameters that shape these ecosystems is essential. This knowledge will enhance our understanding for future wetland design and management.

In recent years, molecular surveys using high-throughput sequencing of 16S ribosomal DNA genes have revealed dynamic changes in soil microbial compositions across various ecosystems [20–22]. Several studies have also used similar approaches to examine the microbial compositions in CWs [20, 23–26]. However, most of these studies have focused on the CWs in temperate regions, leaving microbial compositions and their associated biogeochemical processes in CWs under-documented in other climates. It is certain, however, that tropical and subtropical CWs may exhibit more vigorous biogeochemical reactions due to the increased microbiological activity promoted by warmer temperatures [11, 27–29].

Thus, this study aims to evaluate changes in soil microbial compositions across three subtropical CWs of different ages (i.e., 10, 15, and 17 years at the time of investigation) in northern Taiwan over two different seasons (i.e., winter and summer). The analysis from this study will contribute to a comprehensive understanding of how microbial communities shift over time after wetlands are constructed. We hypothesized that the composition of soil microbial communities in subtropical constructed wetlands for wastewater treatment would differ among wetlands of varying ages and seasons. Additionally, variations in soil nutrient and organic carbon concentrations in constructed wetlands of different ages will lead to differences in nitrification and denitrification-related communities.

## Materials and Methods

### Site Description and Soil Sampling

This study investigated three subtropical CWs in northern Taiwan, where annual precipitation ranges from 2000 to 2500 mm, with increased rainfall during the summer months, despite relatively abundant rainfall year-round. The annual average temperature ranges from 14 to 33 °C (Figure S1). The ages of the CWs were identified based on the time between the soil sampling and each wetland’s completion: Xinhai Phase 1 CW (designated as 17Y; 25° 01′ 57.7″N 121° 27′ 32.1″E), Xinhai Phase 2 CW (designated as 15Y; 25° 01′ 39.5″N 121° 27′ 06.6″ E) and Xinhai Phase 3 CW (designated as 10Y; 25° 01′ 24.2″N 121° 26′ 53.1″E), all located on the right bank of Dahan River in New Taipei City, completed in 2003, 2006, and 2010, respectively. According to previous studies [30, 31], the average water temperature of the Xinhai CW is about 31–38 °C in summer and about 17–20 °C in winter.

Phase 1 CW consists of five treatment cells forming a sequential wetland system connected through gravity flow, with a daily water treatment capacity of around 6000 cubic meters per day (CMD) and covering an area of 7.2 ha. The hydraulic retention time (HRT) of each treatment cell in the CW is about 1.1 days and has an overall HRT of 5.3 days. The biochemical oxygen demand (BOD_5_) pollution reduction rate is greater than 65%, with an outflow concentration of less than 25 mg L^−1^. The ammonia nitrogen (NH_3_-N) pollution reduction rate exceeds 65%, resulting in an outflow concentration of less than 15 mg L^−1^. Additionally, the suspended solids (SS) reduction rate is greater than 65%, with an outflow concentration of less than 15 mg L^−1^ [32].

Similarly, Phase 2 CW is also a wetland system composed of four sequential treatment cells, though it operates at a lower daily water treatment capacity of around 4000 CMD, spans 3.4 ha, and has a designed HRT of 0.9 days per treatment cell and 4.4 days for the whole CW. The pollution reduction rates are as follows: BOD_5_ reduction rate exceeds 60%, with an outflow concentration of less than 30 mg L^−1^; NH_3_-N reduction rate exceeds 60%, with an outflow concentration of less than 20 mg L^−1^; and SS reduction rate exceeds 60%, with an outflow concentration of less than 30 mg L^−1^ [32].

In contrast, Phase 3 CW has an inflow cell, functioning as a forebay, followed by eight parallel treatment cells. It covers a total area of 4.1 ha and has a designed treatment capacity of approximately 5000 CMD. The HRT is designed as 2.8 days for the inflow cell, 0.4 days for the eight parallel cells, and 3.2 days overall for the entire system. BOD_5_ pollution reduction rate is greater than 60%, with an outflow concentration of less than 20 mg L^−1^. The NH_3_-N pollution reduction rate exceeds 50%, resulting in an outflow concentration of less than 15 mg L^−1^. Additionally, the SS pollution reduction rate is greater than 55%, with an outflow concentration of less than 15 mg L^−1^ [32].

Soil samples were collected in August 2020 (summer) and February 2021 (winter) from nine different treatment cells across three CWs, with three cells per CW (Phase 1, Phase 2, and Phase 3, designated as S1-S3 and W1-W3, respectively).

In each treatment cell, one composite soil sample was collected by pooling 15 subsamples taken at spatially distinct points. Sampling followed a random walk procedure in which the sampler started from a randomly chosen edge of the cell and moved along a randomized path, adjusting direction every 5–10 steps (approximately 3–5 m). To avoid localized effects, we intentionally excluded areas near inflow and outflow structures and densely vegetated patches. At each selected point, soil cores were collected using a soil sampler and sliced into two depth intervals (0–2 cm and 2–5 cm) with a sterilized knife. Visible litter and coarse roots were removed before compositing. Samples from each depth interval were collected and stored separately in pre-sterilized sampling bags. All samples were promptly transported to the laboratory and stored at 4 °C for further analysis.

### Soil Physicochemical Property Analyses

Soil pH and electrical conductivity (EC) were measured with 1:1 [33] and 1:5 soil-to-water suspension methods [34]. Soil TOC and total nitrogen (TN) were analyzed using an elemental analyzer (Enviro EL cube, Elementar, Langenselbold, Germany) [35]. Soil water content (SWC) was determined following the method described by Perrier and Salkini [36].

The soil soluble organic C (S_b_OC) and ionic nutrients were extracted using a hot-water extraction method based on previous studies [37]. Briefly, 10 g of soil from each replicate was incubated with a 1:5 soil-to-water ratio at 70 °C for 18 h. Then, this slurry was centrifuged at 1500 rpm for 5 min to collect the suspension (DM0412, DLAB, California, USA). The amounts of NH_4_^+^, nitrate (NO_3_^−^), and sulfate (SO_4_^2−^) in the suspension were measured with an ion chromatograph (Eco IC, Metrohm, Herisau, Switzerland) [38, 39]. In addition, S_b_OC was analyzed using a TOC analyzer (Aurora Model 1030 W, OI Analytical, Texas, USA).

### 16S rRNA Amplicon Sequencing of Constructed Wetland Soils

Total genomic DNA was extracted from 0.5 g of fresh CW soil using a DNA extraction kit (DNeasy PowerSoil kit, QIAGEN GmbH, Hilden, Germany) according to the manufacturer’s instructions. The extracted DNA was stored at − 20 °C for further analysis.

The V4 region of the soil 16S rRNA genes was amplified using the universal primer pair, 515 F (5′-GTGYCAGCMGCCGCGGTAA-3′) and 806R (5′-GGACTACNVGGGTWTCTAAT-3′), through a polymerase chain reaction (PCR) [40]. Each reaction mixture contained 10 µl of Taq Master mix (Taq Master Mix RED reagent kit, AMPLIQON, Odense M, Denmark), 0.4 µl of 515 F primer, 0.4 µl of 806R primer, 8.2 µl of double-distilled water, and 1 µl of extracted DNA, for a total volume of 20 µl. The PCR amplification was conducted with a thermal cycler (MiniAmp Plus, Thermo Fisher Scientific, Waltham, MA, USA) under the following cycling conditions: an initial denaturation at 95 °C for 3 min; 32 cycles of 95 °C for 30 s, 55 °C for 30 s, 72 °C for 30 s; a final extension at 72 °C for 5 min; and a holding step at 4 °C until sample collection. Then, barcode PCR amplification was conducted according to the Illumina Nextera library preparation protocol. Following library construction, the pooled sample was quantified using a fluorometer (Qubit 4 Fluorometer, Thermo Fisher Scientific, Waltham, MA, USA) and corresponding quantification kit (Qubit dsDNA High Sensitivity Assay Kit, Thermo Fisher Scientific, Waltham, MA, USA). Then, the library was sequenced on an Illumina MiSeq platform, with sequencing services provided by the National Genomics Center for Clinical and Biotechnological Applications of the Cancer Progression Research Center, National Yang Ming Chiao Tung University.

### Data Processes and Statistical Analysis

The sequencing data were assembled, and operational taxonomic units (OTUs) were calculated with a 93% sequence similarity threshold (0.07 distance cutoff) using Mothur version 1.47.0 [41] and classified with the SILVA 138.1 database [42]. The OTUs with relative abundance greater than 1% were selected for the construction of phylogenetic trees using MEGA X [43]. PICRUSt2 [44] was used to predict KEGG orthologs from 16S rRNA sequences and to identify key metabolic pathways, including nitrogen, sulfur, and methane metabolism. These predictions were used to support taxonomic inferences regarding functional potential. To explore the potential functional roles of microbial taxa, we referenced the KEGG database to identify representative genera associated with key metabolic modules, such as denitrification, sulfur oxidation, and nitrification. Specifically, we consulted KEGG Modules (e.g., M00529 for denitrification and M00595 for nitrification) and recorded the genera linked to each module. These functional genera were then cross-referenced with the taxonomic table generated from 16S rRNA amplicon sequencing using the SILVA 138.1 database. Genera present in both our dataset and the KEGG module references were identified as potential functional candidates and used as ecological indicators in subsequent analyses. To distinguish between autotrophic and heterotrophic microorganisms in our 16S rRNA dataset, we assigned OTUs to functional groups based on genus-level taxonomic classification and referenced previous literature describing their dominant metabolic strategies.

Alpha and beta diversities of the OTUs were also computed using Mothur. Beta diversity was assessed through principal coordinate analysis (PCoA) based on the Bray–Curtis distance to illustrate the differences among soil communities. Permutational multivariate analysis of variance (PERMANOVA) was performed to analyze whether the community composition between the two depths differs significantly across wetlands and seasons.

The assumptions of normality and homogeneity of variances were tested before analysis of variance (ANOVA) and multiple linear regression (MLR). Normality was assessed using the Shapiro–Wilk test, and homogeneity of variance was verified using Levene’s test. Data that violated the normality assumption were log-transformed, after which they conformed to a normal distribution (Shapiro–Wilk test, *p* > 0.05). After transformations, all data also satisfied the assumption of homogeneity of variances (Levene’s test, *p* > 0.05), confirming their suitability for subsequent statistical analyses. Two-way ANOVA was performed to evaluate the effects of constructed wetland (CW) and season on soil physicochemical properties, alpha diversity, and microbial community structure, with Tukey’s honestly significant difference (HSD) tests used for post hoc pairwise comparisons. Differences between soil depths (0–2 cm and 2–5 cm) were assessed separately using one-way ANOVA, followed by least significant difference (LSD) tests. Analyses were performed using JMP 11.0 (SAS Inc., Cary, NC, USA) with significance set at *p* < 0.05.

Redundancy analysis (RDA) and multiple linear regression (MLR) were used to explore the potential correlation between soil nutrient contents and the relative abundance of OTUs involved in denitrification and nitrification. The analyses were performed with R Studio and the vegan package [45] and explanatory variables with VIF > 10 (variance inflation factor) were removed. The OTU abundances were Hellinger-transformed.

To assess the relationships between denitrifying microbial community composition and environmental variables, Mantel tests were performed. Specifically, Bray–Curtis distance matrices calculated from the relative abundance data of autotrophic and heterotrophic denitrifying microorganisms were correlated with Euclidean distance matrices derived from soil physicochemical parameters. Additionally, Spearman correlation analyses were conducted to examine the relationships among individual soil physical and chemical properties.

All the raw sequences obtained from Illumina MiSeq sequencing have been deposited in the NCBI under the BioProject number: PRJNA1106561 and PRJNA1224543.

## Results

Results showed that soil physicochemical properties such as TOC, TN, S_b_OC, NH_4_^+^, NO_3_^−^, NO_2_^−^, SO_4_^2−^, pH, EC, and SWC, were statistically similar among the three CWs at 0–2 cm soil depths (Table 1; Table S1). Soil NO_3_^−^ concentrations were higher in summer, while NH_4_^+^ concentrations were higher in winter at 2–5 cm soil depths (Table 2; Table S1). In addition, soil TOC, S_b_OC, and EC were higher in the surface soils (i.e., 0–2 cm) than in the subsurface soils (i.e., 2–5 cm).

**Table 1:** Means and standard deviations (mean ± SD) of soil physicochemical properties in studied constructed wetland 0–2 cm soil

Site	Season	TOC (%)	TN (%)	S_b_OC (mg-C kg^−1^ soil)	NO_3_^−^	NO_2_^−^	NH_4_^+^	SO_4_^2−^	pH	EC (µS cm^−1^)	SWC (%)
					(mg kg^−1^ soil)						
Phase 1 (17Y)	Summer	1.8 ± 0.7	0.2 ± 0.04	2.7 ± 1.0	16.5 ± 7.6	7.7 ± 6.6	1.0 ± 0.6	8.1 ± 1.4	6.1 ± 0.7	0.1 ± 0.04	26.3 ± 6.5
	Winter	1.4 ± 0.3	0.1 ± 0.02	0.8 ± 0.3	1.1 ± 1.0	1.1 ± 1.0	1.2 ± 0.7	335.7 ± 384.6	4.7 ± 0.9	0.5 ± 0.4	40.6 ± 6.8
Phase 2 (15Y)	Summer	1.5 ± 0.4	0.2 ± 0.03	0.9 ± 0.4	12.3 ± 6.5	0.1 ± 0.1	2.7 ± 3.7	408.2 ± 90.6	4.4 ± 0.5	0.7 ± 0.2	42.5 ± 9.2
	Winter	3.3 ± 0.9	0.3 ± 0.1	1.2 ± 0.6	0.7 ± 0.6	0.7 ± 0.6	3.1 ± 2.2	152.4 ± 173.0	5.8 ± 0.9	0.5 ± 0.4	49.5 ± 7.6
Phase 3 (10Y)	Summer	1.6 ± 0.5	0.2 ± 0.04	0.9 ± 0.5	9.8 ± 10.9	2.0 ± 1.7	2.1 ± 3.0	209.4 ± 214.0	4.4 ± 0.8	0.4 ± 0.4	39.7 ± 8.6
	Winter	2.0 ± 0.7	0.2 ± 0.1	1.1 ± 0.5	11.5 ± 19.4	11.5 ± 19.4	4.2 ± 4.3	231.6 ± 192.6	4.8 ± 0.7	0.5 ± 0.3	46.2 ± 10.0

*EC* soil electrical conductivity, *TOC* total organic C, *TN* total nitrogen, *S*_*b*_*OC* soluble organic C, *SWC* soil water content

**Table 2:** Means and standard deviations (mean ± SD) of soil physicochemical properties in studied constructed wetland 2–5 cm soil

Site	Season	TOC (%)	TN (%)	S_b_OC (mg-C kg^−1^ soil)	NO_3_^−^	NO_2_^−^	NH_4_^+^	SO_4_^2−^	pH	EC (µS cm^−1^)	SWC (%)
					(mg kg^−1^ soil)						
Phase 1 (17Y)	Summer	1.2 ± 0.4	0.1 ± 0.03	0.7 ± 0.4	11.7 ± 1.0	2.0 ± 1.7	0.1 ± 0.1	8.0 ± 2.6	6.3 ± 0.5	0.1 ± 0.01	23.7 ± 5.1
	Winter	0.8 ± 0.1	0.1 ± 0.01	0.6 ± 0.1	1.0 ± 1.4	0.2 ± 0.2	1.7 ± 2.3	385.2 ± 390.2	3.9 ± 0.3	0.5 ± 0.4	27.2 ± 1.7
Phase 2 (15Y)	Summer	1.2 ± 0.3	0.1 ± 0.03	0.6 ± 0.1	14.2. ± 1.3	0.1 ± 0.1	0.4 ± 0.5	318.7 ± 111.9	3.9 ± 0.3	0.4 ± 0.1	35.1 ± 7.1
	Winter	2.1 ± 0.6	0.2 ± 0.04	0.8 ± 0.2	3.0 ± 2.5	0.2 ± 0.1	2.3 ± 0.3	142.1 ± 244.2	5.8 ± 0.9	0.5 ± 0.4	42.2 ± 8.9
Phase 3 (10Y)	Summer	1.0 ± 0.3	0.1 ± 0.04	0.4 ± 0.2	5.7 ± 4.5	0.5 ± 0.7	0.1 ± 0.1	145.9 ± 144.5	4.6 ± 1.1	0.2 ± 0.2	34.7 ± 8.5
	Winter	1.8 ± 0.6	0.2 ± 0.1	0.5 ± 0.2	4.4 ± 7.4	0.1 ± 0.2	1.7 ± 2.1	256.3 ± 285.6	4.8 ± 0.7	0.5 ± 0.3	46.8 ± 9.6

*EC* soil electrical conductivity, *TOC* total organic C, *TN* total nitrogen, *S*_*b*_*OC* soluble organic C, *SWC* soil water content

The NGS results showed that sequence counts for each sample ranged from 45,833 to 124,757. Rarefaction curves indicated that the sequencing depth adequately represented the microbial communities in the collected samples (Figure S2).

**Fig. 1:**
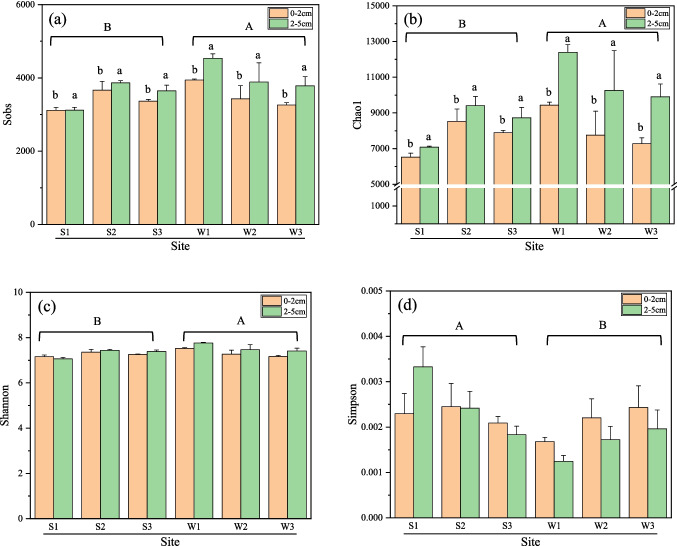
Alpha diversity indices Sobs (**a**), Chao1 (**b**), Shannon (**c**), and Simpson (**d**) of microbial communities in two depths of three constructed wetland soils in Taiwan. (S: Summer; W: Winter; 1–3: Phase 1–3 of the constructed wetlands). Statistical differences were analyzed using two-way ANOVA (CW × season) with Tukey’s HSD post hoc tests and one-way ANOVA (depth) with LSD tests. Uppercase letters (A, B) indicate significant seasonal differences, and lowercase letters (a, b) indicate significant depth-related differences (*p* < 0.05). No significant differences were observed among CWs. Detailed statistical results are provided in Table S2

The ANOVA results indicated no significant differences in alpha diversity among the three CWs. However, the Sobs, Chao1, and Shannon indices were significantly higher in winter than in summer (*p* < 0.001; *p* < 0.001;* p* < 0.01), whereas the Simpson index was significantly higher in summer than in winter (*p* < 0.01). Regarding the spatial distribution, the 2–5 cm layer showed significantly higher values for Sobs, Chao1, and Shannon than the 0–2 cm layer (*p* < 0.001; *p* < 0.001; *p* < 0.001) (Table S2, Fig. 1).

**Fig. 2:**
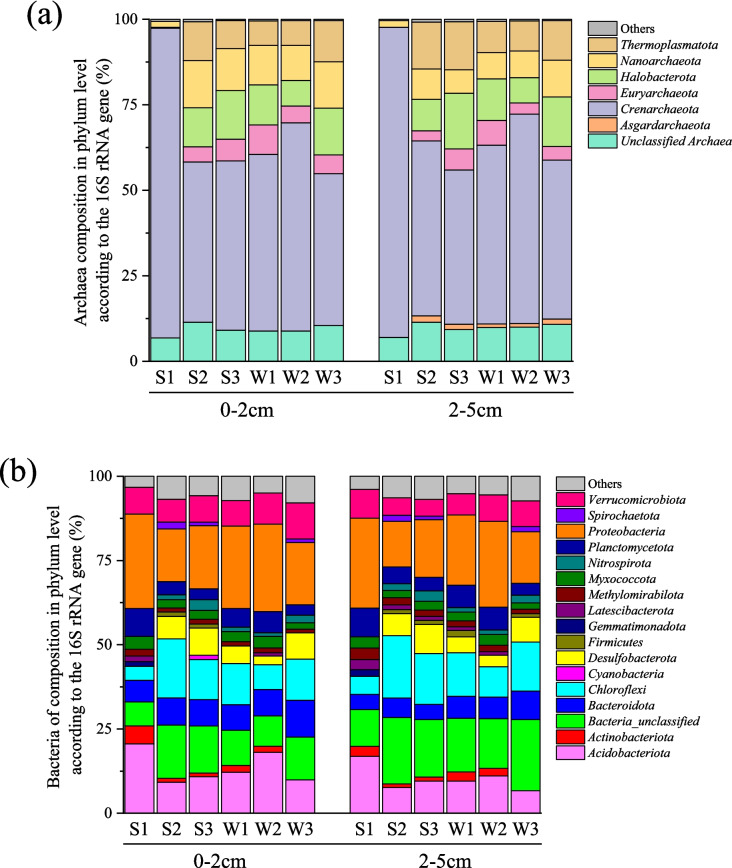
The community compositions of archaea (**a**) and bacteria (**b**) were identified at the phylum level in three constructed wetland soils, with triplicate samples taken at two different depths. (S: Summer; W: Winter; 1–3: Phase 1–3 constructed wetland)

Regarding microbial community composition, archaea constituted 7.8% of the microbial population in the 0–2 cm soil layer and 10.8% in the 2–5 cm layer, while bacteria accounted for 90.8% and 89.1%, respectively (Fig. 2). In S1, the *Crenarchaeota* phylum dominated the archaeal community at both depths (90.5%, 90.7%), followed by other phyla such as unclassified Archaea, *Asgardarchaeota*, *Euryarchaeota*, *Halobacterota*, *Nanoarchaeota*, and *Thermoplasmatota*. Similarly, *Proteobacteria* phyla were highly abundant in the bacterial community in S1 (*p* < 0.01), while *Desulfobacterota*, *Nitrospirota*, *Firmicutes*, and *Spirochaetota* were significantly lower (*p* < 0.05).

**Fig. 3:**
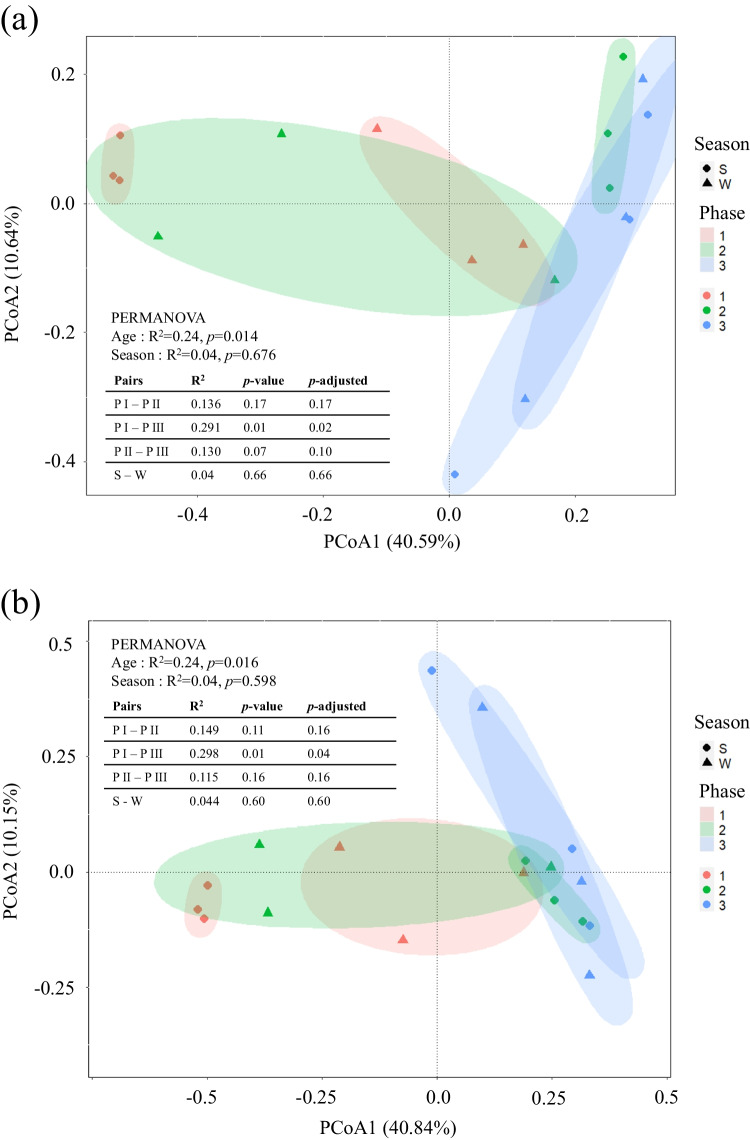
Principal coordinates analysis (PCoA) and PERMANOVA of microbial community in three constructed wetland soils in 0–2 cm (**a**) and 2–5 cm (**b**)

PCoA-based beta diversity analysis using Bray–Curtis distances showed that PC1 and PC2 accounted for 40.59% and 10.64% of the variance in microbial community composition at the 0–2 cm soil depth, and 40.84% and 10.15% at the 2–5 cm depth, respectively (Fig. 3). Despite the similar soil physicochemical properties among the three CWs, the microbial compositions were distinct among the three sites. PERMANOVA results further indicated significant differences between wetlands of different ages (*p* < 0.05), with pairwise analysis showing significant differences between Phase 1 and Phase 3 (*p* < 0.05), though no significant differences were observed between seasons (*p* > 0.05). Consistent with the PCoA results, the heatmap of the first 20 OTUs also revealed distinct microbial compositions in S1 at the 0–2 cm and 2–5 cm soil depths. The OTU 1 was more abundant in 0–2 cm and 2–5 cm soils of CWs other than S1, where OTU 2 and OTU 6 were more prevalent (Figure S3).

Phylogenetic analysis of microbes with OTUs showing relative abundances greater than 1% in the surface soils (i.e., 0–2 cm) indicated that OTU 1 was closely related to ammonia-oxidizing archaea, *Nitrososphaera*. However, OTU 2 and OTU 6 were respectively associated with *Limisphaera* and *Luteitalea* (Figure S4).

Phylogenetic analysis of microbes at the 2–5 cm soil depths indicated that OTUs 1, 5, 22, 48, 55, 57, and 70 were distinctively related to ammonia-oxidizing archaea, while OTU 2 and OTU 4 were associated with *Limisphaera* and *Luteitalea*, respectively (Figure S5). Furthermore, hierarchical clustering of nearest neighbor distances revealed stronger clustering patterns in Phase 1 CW samples, while Phase 3 samples displayed greater variability at both 0–2 cm and 2–5 cm soil depths. In addition, phylogenetic analysis of microbial communities in CW soils showed that most OTUs at 0–2 and 2–5 cm soil depths were related to nitrifying and denitrifying microorganisms, while others were associated with microbes involved in sulfur oxidation and iron reduction functions.

**Fig. 4:**
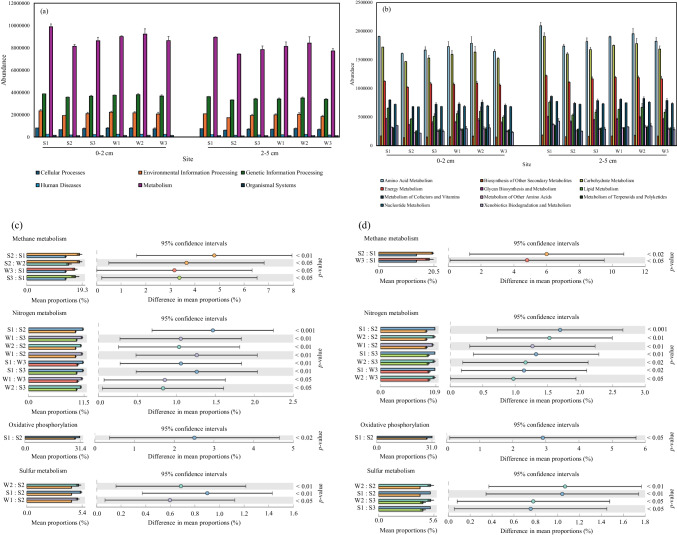
The results of KEGG annotations functional pathway predicted by PICRUSt2 based on 16S rRNA gene sequencing data of three CWs in summer and winter in KEGG level 1 (**a**) and KEGG level 2 of “Metabolism” (**b**) in two depths. The KEGG level 3 of “Energy Metabolism” between six groups (S1, S2, S3, W1, W2, W3) in the 0–2 cm soil layer (**c**) and the 2–5 cm soil layer (**d**) was analyzed by ANOVA and Tukey’s HSD test with a significant level of 0.05 (*p* < 0.05)

To support functional inference based on taxonomy, PICRUSt2 was used to predict KEGG metabolic pathways from 16S rRNA data. At KEGG Level 1, “Metabolism” was the most abundant category across all sites and soil depths, followed by “Genetic Information Processing” and “Environmental Information Processing” (Fig. 4a). Within Level 2 of “Metabolism”, the top pathways included energy metabolism, carbohydrate metabolism, and amino acid metabolism (Fig. 4b). Among these, energy metabolism was selected for further analysis due to its ecological specificity. While carbohydrate and amino acid metabolism were abundant, they represent general cellular functions. In contrast, energy metabolism includes sub-pathways directly involved in biogeochemical transformations critical to wastewater treatment, such as nitrification, denitrification, sulfur oxidation, and methane oxidation. At Level 3, significant differences (*p* < 0.05) were observed in nitrogen metabolism, sulfur metabolism, methane metabolism, and oxidative phosphorylation across sites and seasons (Fig. 4c, d). Among them, nitrogen metabolism showed the most consistent and pronounced variation across both depths and wetland ages, indicating its sensitivity to environmental and operational differences. Given its central role in nutrient removal, nitrogen metabolism is highlighted as a potential key functional target for future mechanistic and gene-based studies in CWs.

**Fig. 5:**
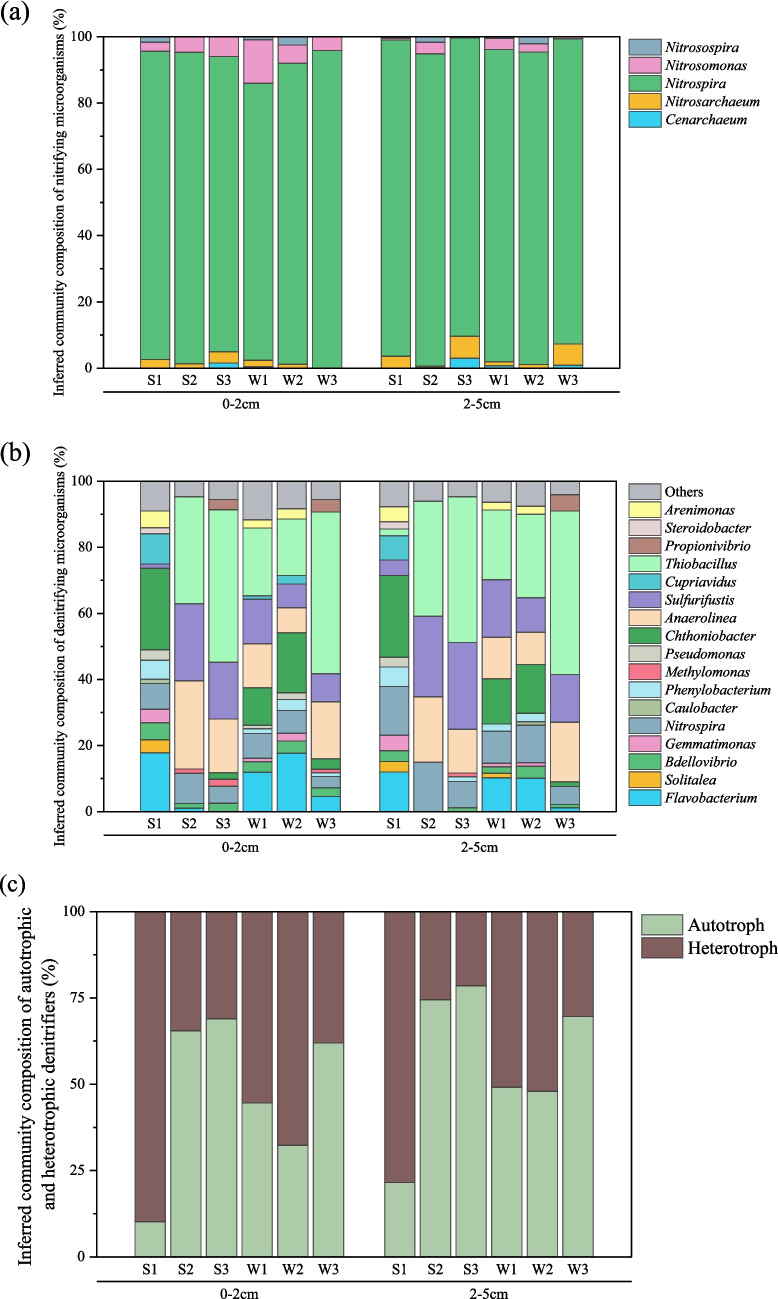
Inferred community composition of nitrifying microorganisms (**a**), denitrifying microorganisms (**b**), and autotrophic and heterotrophic denitrifiers (**c**) in soils from three constructed wetlands at two depths. Functional assignments are based on genus-level taxonomy and KEGG database annotations

To further explore microbial communities potentially involved in nitrogen cycling, we analyzed the relative abundances of ammonia-oxidizing archaea, ammonia-oxidizing bacteria, and denitrifying bacteria based on 16S rRNA sequencing data and functional annotations from the KEGG database (Fig. 5). The ammonia-oxidizing community showed similar composition at both depths, with the genus *Nitrospira* accounting for over 90% of the relative abundance, followed by *Nitrosomonas* and *Nitrosarchaeum*.

The denitrifying genera, including *Thiobacillus*, *Sulfurifustis*, *Anaerolinea*, and *Nitrospira*, dominated the CW soils, except in S1, where *Chthoniobacter* and *Flavobacterium* exhibited the highest relative abundance. The relative abundance of denitrifying bacteria was further categorized into autotrophic and heterotrophic groups and analyzed using two-way ANOVA. The results revealed a significant difference in the composition of autotrophic and heterotrophic denitrifying bacteria among the three CWs (*p* < 0.05). Autotrophic denitrifying bacteria were significantly more abundant in younger CWs compared to older CWs (i.e., Phase 3 ≥ Phase 2 > Phase 1) at both soil depths and during both sampling seasons. Additionally, the abundance of autotrophic denitrifying bacteria was higher at the 2–5 cm soil depth than at the 0–2 cm (*p* < 0.05).

**Fig. 6:**
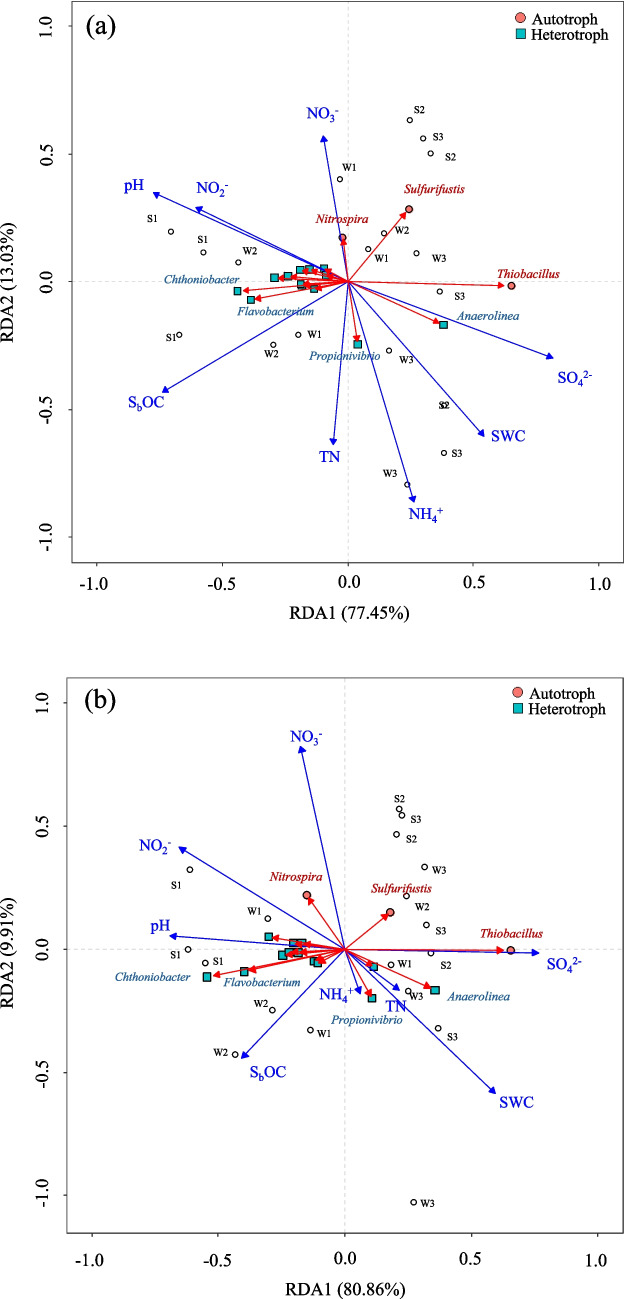
Redundancy analysis (RDA) between denitrifiers and soil physicochemical properties in the three constructed wetland soils at 0–2 cm (**a**) and 2–5 cm (**b**) depths. The autotrophs are labeled with red circles; the heterotrophs are labeled with blue squares. [S_b_OC: soluble organic C; NH_4_^+^: ammonium; NO_3_.^−^: nitrate; EC: soil conductivity; SWC: soil water content]

Mantel test results indicated that both autotrophic and heterotrophic denitrifying bacteria were significantly correlated with pH, S_b_OC, SO_4_^2−^, NO_2_,^−^ and SWC at both soil depths (Figure S6). The RDA showed that soil physicochemical factors differently affected autotrophic and heterotrophic denitrifying microorganisms (Fig. 6). Autotrophic denitrifying bacteria are correlated with nutrient concentrations including NO_3_^−^, NO_2_^−^, and SO_4_^2−^. The genus *Nitrospira* shows a potential relationship with NO_3_^−^ and NO_2_^−^ concentrations, while *Sulfurifustis* and *Thiobacillus* are linked to SO_4_^2−^ concentrations. Most heterotrophic denitrifying bacteria are associated with factors such as pH, S_b_OC, and TN.

Furthermore, MLR analysis revealed that most heterotrophic denitrifying bacteria had a significant positive correlation with S_b_OC and pH, along with a significant negative correlation with NH_4_^+^ concentrations at the 0–2 cm soil depths (Tables S3, S4). In contrast, autotrophic denitrifying bacteria showed a significant positive correlation with NO_3_^−^ and NH_4_^+^ concentrations, but a significant negative correlation with S_b_OC concentrations at the same soil depths.

At the 2–5 cm soil depth, a significant positive correlation between heterotrophic denitrifying bacteria and S_b_OC, as well as NO_2_^−^ concentrations, was observed, along with a negative correlation with SO_4_^2−^ concentrations. Meanwhile, autotrophic denitrifying bacteria, such as *Nitrospira,* showed a significant positive correlation with NO_3_^−^ concentrations, while *Thiobacillus* was positively correlated with SO_4_^2−^ concentrations and negatively correlated with S_b_OC concentrations.

## Discussion

### Soil Physicochemical, Microbial Diversity, and Richness in Two Depths of Different Constructed Wetlands

This study provides a comprehensive evaluation of soil microbial composition and its potential relationships to soil physicochemical properties. The results indicate that most soil physicochemical factors did not significantly differ across the CWs studied. However, S_b_OC and TOC in the 0–2 cm soil layer were significantly higher than in the 2–5 cm layer, suggesting that the topsoil is an important zone for carbon accumulation in CWs [46, 47].

The observed higher alpha diversity in the CWs during winter compared to summer, as well as higher diversity in subsurface soil than surface soil, may be attributed to elevated summer water temperatures warming surface soils exposed to sunlight, potentially inhibiting the soil microbial community [48, 49]. Furthermore, the higher S_b_OC in the 0–2 cm soil layer compared to the 2–5 cm layer may favor the growth of r-selected microorganisms [50, 51]. A similar trend was also observed in Phase 1, where elevated S_b_OC levels in surface soils in summer corresponded to both reduced alpha diversity and increased heterotrophic abundance. These findings suggest a potential shift in community structure toward dominance by a few taxa under labile carbon-enriched conditions, as also supported by previous studies [52, 53]. Seasonal hydrological conditions, such as increased rainfall or prolonged water saturation, may also influence microbial community composition by altering oxygen or nutrient availability [54–57]. While our findings showed the potential role of temperature, unmeasured environmental variables in this study may have also contributed to the observed seasonal variation. Future studies could explore the combined effects of temperature, hydrology, and nutrient dynamics in more detail.

Despite similar soil physicochemical properties among the three studied CWs, the relative abundance at the phylum level and PERMANOVA analysis of the Bray–Curtis distance matrix for OTUs both indicated a significant influence of wetland age and soil depth on community structure, corroborating the findings of previous studies [58–61]. Additionally, the PCoA results revealed that older CWs exhibited greater shifts in microbial community composition between winter and summer compared to younger CWs. However, within the same season, microbial communities from replicate sampling sites in older CWs were more similar to each other than those in younger CWs. This pattern suggests that while older wetlands may experience greater seasonal variability in microbial composition, they also exhibit increased spatial homogeneity within seasons, indicating a form of community stabilization over time. Such stabilization could be attributed to the development of more uniform environmental conditions and the establishment of microbial networks in mature wetlands [14, 19].

The predominant archaeal abundance across all CWs was composed primarily of the phylum *Crenarchaeota*, also referred to as *Thaumarchaeota* or *Thermoproteota* [62, 63]. *Crenarchaeota* is widely found in wetlands and other soil environments [64–67] and plays a crucial role in ammonia oxidation within both natural and constructed wetland ecosystems [68–70]. Therefore, we suspect that their abundance may contribute to ammonia oxidation processes in the studied CWs. Furthermore, the highest relative abundance of *Crenarchaeota* in the S1 may explain the observed lowest average NH_4_^+^ concentration and highest NO_3_^−^ and NO_2_^−^ concentrations at that site.

The *Halobacterota* and *Thermoplasmatota* phyla were the second- and third-most abundant archaeal groups in the studied CWs, with the exception of S1. These groups include methanogenic archaea, as reported in previous studies [71, 72], suggesting their potential involvement in methane-related processes within the system. Similarly, the order *Woesearchaeales*, belonging to the phylum *Nanoarchaeota*, was consistently identified as the dominant archaeal group across all sites. Although its specific ecological role remains unclear, previous research suggests a potential symbiotic relationship with methanogenic archaea [73], which may influence microbial interactions within the studied CWs. Further investigation of methane flux in CWs will be valuable in clarifying the link between these archaeal groups and methanogenesis.

The predominant bacterial phyla—*Proteobacteria*, *Chloroflexi*, and *Acidobacteriota*—observed in the studied CWs are commonly found in both constructed wetlands and natural wetlands due to their essential roles in carbon and nitrogen cycling within these ecosystems [20, 67, 74–79].

Another significant phylum, *Bacteroidota,* primarily represented by the *Bacteroidia* class, includes many denitrifying bacteria [80] and was also prominent in the CW soils. Similarly, the anaerobic ammonia-oxidizing bacterium *Candidatus_Anammoximicrobium*, previously classified within the phylum *Planctomycetota* [81], was also detected in the collected samples. Bacteria from the phylum *Desulfobacterota*, mainly involved in sulfate reduction, showed a significantly lower relative abundance at site S1, possibly due to the low average SO_4_^2−^ concentrations observed at this site.

Overall, the microbial compositions of CW soils suggest microbial functions are primarily centered on nitrification and denitrification processes in the studied sites, regardless of their age or seasonal variation.

### Abundance of Nitrifying and Denitrifying Microorganisms in Constructed Wetlands

The phylogenetic tree of OTUs with a relative abundance exceeding 1% revealed that most OTUs were distinctly related to bacteria involved in nitrification, denitrification, and sulfur oxidation [82–90]. For example, OTU 1, which exhibited the highest relative abundance in both the 0–2 cm and 2–5 cm soil depths, was related to the *Nitrososphaera viennensis* and *Nitrosopumilus piranensis*, key archaea involved in regulating the soil nitrification processes [91, 92]. Meanwhile, OTU 2, dominant in the 2–5 cm soil layer, showed a distinct relationship to *Luteitalea pratensis*. According to the KEGG GENOME database, *Luteitalea pratensis* possesses a nitrate reduction pathway, which potentially contributes to denitrification processes [93]. Across all groups, the microbial communities were dominated by *Nitrospira* (Fig. 5a), a diverse and abundant group of nitrite-oxidizing bacteria (NOB) found in natural habitats such as soils, sediments, and oceans, as well as in wastewater treatment plants [94, 95].

In the studied CWs, several sulfur-related autotrophic denitrifiers were identified, specifically the genera *Thiobacillus* and *Sulfurifustis*. Notably, the genus *Thiobacillus* was particularly abundant in S2, S3, and W3, with a relative abundance ranging from 32.3 to 49.5% in the 0–2 cm and 2–5 cm soil layers. The genus *Sulfurifustis* was also prevalent in the 0–2 cm soil layer, with a relative abundance of 23.3% to 26.2% (Fig. 5b). These genera have been associated with sulfur-driven autotrophic denitrification in various environments, including CWs [96–99], and their high abundance in this study suggests that sulfur cycling may substantially contribute to nitrogen removal. This interpretation is consistent with findings by Burgin and Hamilton [100], who reported that sulfur-oxidizing bacteria are widespread in freshwater ecosystems and can facilitate nitrate removal through autotrophic denitrification, even under low sulfur availability. The presence of sulfur compounds introduced via wastewater inputs may further enhance this pathway in CWs, potentially shifting the balance between autotrophic and heterotrophic denitrification processes. While our interpretations are based on 16S rRNA gene data, the observed microbial composition may suggest a possible coupling of nitrogen and sulfur transformations. Future studies using functional gene expression analysis or stable isotope tracing would help confirm the contribution of these pathways.

The proportion of heterotrophic denitrifying bacteria significantly increases with the age of the CWs, possibly due to the accumulation of soil carbon sources over time. Studies have indicated that hot-water-extracted S_b_OC is considered a readily metabolizable carbon source for microbes [37, 101]. The increase in S_b_OC may enhance the relative abundance of heterotrophic denitrifying bacteria over autotrophic denitrifiers, likely due to greater availability of organic carbon as an energy source. Similar patterns have been reported in previous studies [102, 103], where increased dissolved or bioavailable organic carbon inputs shifted denitrifier communities toward heterotrophic dominance. Conventional wastewater treatment has primarily relied on heterotrophic denitrification, which often struggles to maintain high NO_3_^−^ removal efficiency when organic carbon sources are insufficient [102, 104, 105]. However, autotrophic denitrification, driven by sulfur oxidation, has been implemented for NO_3_^−^ removal from wastewater in CWs [106, 107], with a similar distribution observed in this study. Further research is needed to clarify the importance of autotrophic denitrification in regulating the nutrient dissipation in young CWs, where insufficient carbon accumulation may limit the efficiency of heterotrophic denitrification.

In addition, synergistic microbial pathways for nitrogen removal have received increasing attention in CWs in recent years [22, 108]. Previous studies have emphasized the high pollutant removal efficiency of heterotrophic and autotrophic synergistic denitrification systems, highlighting the complex bacterial interactions on nitrogen removal in the system [109]. Clearly, nitrogen removal in CWs is achieved through multiple pathways, including autotrophic nitrification, heterotrophic denitrification, autotrophic denitrification, and possibly anaerobic ammonia oxidation. The abundance, community structure, and distribution of these microorganisms are critical factors influencing the performance of CWs. While the taxonomic composition derived from 16S rRNA sequencing provides valuable insights into microbial community structure, we acknowledge the limitations of inferring functional capacity solely from taxonomic data.

### Influence of Environmental Factors to the Microbial Communities in Constructed Wetlands

The Mantel test results demonstrated the key factors such as pH, S_b_OC, SO_4_^2−^, NO_2_,^−^ and SWC significantly influenced the composition of denitrifying bacterial communities in CWs, aligning with the findings from the RDA and MLR results. As previously mentioned, S_b_OC is a major factor affecting the denitrifying bacterial communities, particularly the heterotrophic denitrifiers [110–112]. This relationship is further elucidated by the results of the RDA and MLR, where most heterotrophic denitrifying bacteria exhibited a positive correlation with S_b_OC (Fig. 6, Table S3, Table S4). This was particularly evident in the genus *Chthoniobacter*, which has been associated with organic matter decomposition [113, 114].

Additionally, the high average SO₄^2^⁻ concentrations observed in the CWs suggest that pH and SO₄^2^⁻ played a significant role in the elevated relative abundance of sulfur-autotrophic denitrifying bacterial communities. Previous studies have indicated that acidic pH conditions may influence the efficiency of sulfur-based autotrophic denitrification [115, 116]. The MLR results further revealed that heterotrophic denitrifying bacterial communities were predominantly negatively correlated with SO₄^2^⁻, suggesting ecological niche differentiation between sulfur-autotrophic and heterotrophic denitrifiers.

In this study, the concentration of NO₂⁻ was positively correlated with heterotrophic denitrifying bacteria, and the effect was more pronounced in the 2–5 cm soil layer compared to the 0–2 cm layer. One possible explanation is that the accumulation of NO₂⁻ in subsurface soils may create favorable conditions for heterotrophic denitrifiers, as NO₂⁻ serves as an intermediate electron acceptor in denitrification, particularly under low O₂ conditions. Additionally, pH plays a critical role in denitrification efficiency, as it influences enzyme activity and microbial community composition. A slightly acidic to neutral pH has been shown to enhance NO₂⁻ reduction by stabilizing the activity of key denitrification enzymes, whereas highly acidic conditions can impair N₂O reductase, leading to incomplete denitrification and increased N₂O emissions [117, 118]. The findings suggest that heterotrophic denitrifiers in the deeper soil layers may preferentially utilize the NO₂⁻ pathway for nitrogen removal due to its lower C requirements, reduced O₂ demand, and enhanced denitrification efficiency, as reported in previous studies [119, 120].

Overall, this study highlights the potential ecological preference of denitrifying bacterial communities in CWs. In particular, adjusting the relative availability of C/N/S in influent water or substrates may help the development of microbial communities toward specific denitrification pathways [121, 122]. For example, incorporating sulfate-rich substrates has the potential to enhance sulfur-based autotrophic denitrification, particularly in systems where organic carbon is limited [123, 124]. Conversely, the addition of biodegradable carbon sources, including plant litter or acetate, can support heterotrophic denitrifiers in carbon-deficient conditions [123]. Integrating both autotrophic and heterotrophic pathways within mixotrophic CW systems could potentially improve resilience and nitrogen removal efficiency under fluctuating environmental conditions [107, 125]. These strategies, however, should be tailored to local water chemistry, vegetation structure, and hydraulic dynamics. Future studies may explore the effectiveness of these strategies using long-term monitoring and simulation tools, thereby contributing to improved microbial-based design of CW systems.

## Conclusion

This study provides valuable insights into the composition of microbial communities in CWs, which varied among CWs of different ages and across seasons. Older CWs exhibited more pronounced seasonal shifts in microbial composition, particularly denitrifying bacterial communities, between winter and summer. The results also highlight the significance of environmental factors such as S_b_OC, pH, SO_4_^2−^, and soil moisture in regulating microbial community structure and activity. These factors influence the relative abundance and function of sulfur-autotrophic and heterotrophic denitrifiers, shaping the overall denitrification processes within CWs. These findings serve as a useful reference for informing future CW management strategies. Future research may benefit from integrating functional gene analyses with detailed water quality monitoring to further support the development of CW systems to improve treatment performance and ecological sustainability.

## Supplementary Information

Below is the link to the electronic supplementary material.

Supplementary file1 (DOCX 24321 KB)

## Data Availability

All the raw sequences obtained from Illumina MiSeq sequencing have been deposited in the NCBI under the BioProject number: PRJNA1106561 and PRJNA1224543.
