# Supplementary material for: Effects of Soil Properties and Seasonal Variations on Microbial Communities in Constructed Wetlands
Source: Microb Ecol. 2025 Jun 12;88(1):64. doi: 10.1007/s00248-025-02564-7 (PMC12158856; doi:10.1007/s00248-025-02564-7)
Supplement: Supplementary file 1 — Supplementary file1 (DOCX 24321 KB) [file 248_2025_2564_MOESM1_ESM.docx]

**Table S1** Comparisons of soil physicochemical properties in different sites and season based on two-way ANOVA and Tukey's honestly significant difference (HSD) test with significance level of 0.05 (*p* < 0.05). Two depths of soils are also compared based on one-way ANOVA and least significant difference (LSD) tests with significance level of 0.05 (*p* < 0.05).

|  | 0-2 cm | | |  | 2-5 cm | | |  | Depth effect |
| --- | --- | --- | --- | --- | --- | --- | --- | --- | --- |
|  | Site effect | Season effect | Site$\times$Season |  | Site effect | Season effect | Site$\times$Season |  |  |
| TOC | n.d. | n.d. | n.d. |  | n.d. | n.d. | W2≥W3=S1,2,3≥W1 |  | 0-2 cm > 2-5 cm |
| TN | n.d. | n.d. | n.d. |  | n.d. | n.d. | n.d. |  | n.d. |
| S_b_OC | n.d. | n.d. | S1≥W2=W3≥S2=S3=W1 |  | n.d. | n.d. | n.d. |  | 0-2 cm > 2-5 cm |
| NO_3_^-^ | n.d. | n.d. | n.d. |  | n.d. | S>W | n.d. |  | n.d. |
| NO_2_^-^ | n.d. | n.d. | n.d. |  | n.d. | n.d. | n.d. |  | n.d. |
| NH_4_^+^ | n.d. | n.d. | n.d. |  | n.d. | W>S | n.d. |  | n.d. |
| SO_4_^2-^ | n.d. | n.d. | n.d. |  | n.d. | n.d. | n.d. |  | n.d. |
| pH | n.d. | n.d. | S1≥W2≥W3≥W1≥S2=S3 |  | n.d. | n.d. | S1≥W1,2,3=S3≥S2 |  | n.d. |
| EC | n.d. | n.d. | n.d. |  | n.d. | n.d. | n.d. |  | 0-2 cm > 2-5 cm |
| SWC | n.d. | W>S | n.d. |  | 3=2>1 | W>S | n.d. |  | n.d. |

Note that no differences (n.d.).

[EC: soil electrical conductivity; TOC: total organic C; TN: total nitrogen; S_b_OC: soluble organic C; SWC: soil water content; S: Summer; W: Winter; 1-3: Phase 1-3 constructed wetland].

**Table S2** Comparisons of alpha diversity in different sites and season based on two-way ANOVA and Tukey's honestly significant difference (HSD) test with significance level of 0.05 (*p* < 0.05). Two depths of soils are also compared based on one-way ANOVA and least significant difference (LSD) tests with a significance level of 0.05.

|  | Season effect | Site effect | Season$\times$Site | Depth effect |
| --- | --- | --- | --- | --- |
| Sobs | Winter > Summer | n.d. | W1>S2=W2=W3=S3>S1 | 2-5 cm > 0-2 cm |
| Chao1 | Winter > Summer | n.d. | W1>S2=W2=W3=S3>S1 | 2-5 cm > 0-2 cm |
| Shannon | Winter > Summer | n.d. | W1>S2=W2=W3=S3>S1 | 2-5 cm > 0-2 cm |
| Simpson | Summer > Winter | n.d. | S1≧S2≧W3≧W2=S3≧W1 | n.d. |

Note that no differences (n.d.).

**Table S3** The positive (+) and negative (-) correlations identified through multiple linear regression (MLR) backward elimination between autotrophic and heterotrophic denitrifiers and the physicochemical properties of soil in the 0-2 cm soil at the significant level of 0.05. [S_b_OC: soluble organic C; SWC: soil water content].

|  |  | NO_3_^-^ | NO_2_^-^ | SO_4_^2-^ | NH_4_^+^ | S_b_OC | TN | pH | SWC |
| --- | --- | --- | --- | --- | --- | --- | --- | --- | --- |
| **Autotrophic** | *Nitrospira* |  |  |  |  |  | - |  |  |
|  | *Sulfurifustis* |  |  |  |  | - |  |  |  |
|  | *Thiobacillus* | + |  |  | + | - |  |  |  |
| **Heterotrophic** | *Anaerolinea* |  |  | + |  |  |  |  |  |
|  | *Arenimonas* |  |  |  |  | + |  | + |  |
|  | *Bdellovibrio* |  |  |  |  |  |  | + |  |
|  | *Chthoniobacter* |  |  |  | - | + |  |  |  |
|  | *Cupriavidus* |  | + |  |  |  | + |  |  |
|  | *Flavobacterium* |  | - |  |  | + |  | + |  |
|  | *Gemmatimonas* | - | + | - |  |  |  |  |  |
|  | *Phenylobacterium* |  |  | - |  | + |  |  |  |
|  | *Propionivibrio* |  |  |  | + |  |  |  |  |
|  | *Pseudomonas* |  |  |  |  |  | + |  | - |
|  | *Solitalea* |  | - |  |  | + | - | + |  |
|  | *Sphingomonas* |  | + |  |  |  |  |  |  |
|  | *Steroidobacter* |  |  |  |  | + |  | + |  |

**Table S4** The positive (+) and negative (-) correlations identified through multiple linear regression (MLR) backward elimination between autotrophic and heterotrophic denitrifiers and the physicochemical properties of soil in the 2-5 cm soil at the significant level of 0.05. [S_b_OC: soluble organic C; SWC: soil water content].

|  |  | NO_3_^-^ | NO_2_^-^ | SO_4_^2-^ | NH_4_^+^ | S_b_OC | TN | pH | SWC |
| --- | --- | --- | --- | --- | --- | --- | --- | --- | --- |
| **Autotrophic** | *Nitrospira* | + |  |  |  |  |  |  |  |
|  | *Sulfurifustis* |  |  |  |  |  |  |  |  |
|  | *Thiobacillus* |  |  | + |  | - | + |  |  |
| **Heterotrophic** | *Anaerolinea* |  | - | + |  |  |  |  |  |
|  | *Arenimonas* |  |  | - |  |  | - |  |  |
|  | *Bdellovibrio* |  |  | - |  |  |  |  |  |
|  | *Chthoniobacter* |  |  | - |  |  |  |  |  |
|  | *Cupriavidus* |  | + |  |  | + |  |  |  |
|  | *Flavobacterium* |  |  | - |  | + | - |  |  |
|  | *Gemmatimonas* |  | + |  |  | + |  |  |  |
|  | *Microvirgula* |  |  |  | - |  | + | - |  |
|  | *Phenylobacterium* |  | + | - |  |  |  |  |  |
|  | *Propionivibrio* |  |  |  | - |  |  |  | + |
|  | *Pseudomonas* |  | + |  |  | + |  |  |  |
|  | *Solitalea* |  |  |  |  | + |  |  | - |
|  | *Steroidobacter* |  |  |  |  |  |  | + |  |

**Figure S1** The three constructed wetlands (Phases 1, 2, and 3) of the study in Banqiao, New Taipei City, Taiwan. The green borders spotted the location of three constructed wetlands. Green borders indicate the boundaries of each constructed wetland, and pink triangles represent the treatment cells where soil sampling was conducted. Sampling areas were selected to avoid inlet and outlet zones to reduce the influence of localized hydraulic effects.

**Figure S2** Rarefaction curve of the 16S rRNA genes of 0-2 cm soil (a) and 2-5 cm soil (b), calculated at the OTU level with a 93% sequence similarity threshold (0.07 distance cutoff).

**Figure S3** Heatmap showing the relative abundance of OTU1 to OTU20 in soils from three constructed wetlands at two depths: 0–2 cm (a) and 2–5 cm (b). Abundance values were normalized to relative abundance (%) and visualized using a color gradient, where darker red indicates higher abundance and lighter colors indicate lower abundance (color key shown in the top left of each panel). Samples are grouped by season along the top color bar: blue indicates summer, and orange indicates winter. Hierarchical clustering was performed using Bray–Curtis dissimilarity with the nearest neighbor method. The resulting dendrograms (top of each panel) illustrate compositional similarities among samples, facilitating interpretation of seasonal shifts in microbial communities.

**Figure S4** The maximum likelihood phylogenetic tree illustrates the 16S rRNA lineages identified in the 0–2 cm soil layer of the three constructed wetlands. Bootstrap values above 50% are shown at the corresponding nodes. Only OTUs related to denitrifying microorganisms with a relative abundance larger than 1.0% in at least one sample are included in the phylogenetic tree.

**Figure S5** The maximum likelihood phylogenetic tree illustrates the 16S rRNA lineages identified in the 2-5 cm soil layer of the three constructed wetlands. Bootstrap values above 50% are shown at the corresponding nodes. Only OTUs related to denitrifying microorganisms with a relative abundance larger than 1.0% in at least one sample are included in the phylogenetic tree.

**Figure S6** Mantel test results showing correlations between environmental variables and the composition of autotrophic and heterotrophic denitrifiers in surface (0–2 cm) (a), and subsurface (2–5 cm) (b) soils. Orange lines represent p-values < 0.01 and green lines represent p-values < 0.05. Line thickness reflects the strength of correlation, with thicker lines indicating higher Mantel r values. [S_b_OC: soluble organic C; SWC: soil water content].
